# Ascites and Serial Plasma Circulating Tumor DNA for Predicting the Effectiveness of Hyperthermic Intraperitoneal Chemotherapy in Patients With Peritoneal Carcinomatosis

**DOI:** 10.3389/fonc.2022.791418

**Published:** 2022-01-25

**Authors:** Xiaolin Pu, Zongyuan Li, Xiaoying Wang, Hua Jiang

**Affiliations:** ^1^Department of Oncology, The Affiliated Changzhou No. 2 People’s Hospital of Nanjing Medical University, Changzhou, China; ^2^Department of Oncology, Graduate School of Dalian Medical University, Dalian, China

**Keywords:** circulating tumor DNA, hyperthermic intraperitoneal chemotherapy, peritoneal carcinomatosis, variant allele frequency, molecular tumor burden index

## Abstract

**Purpose:**

We investigated the value of ascites and serial plasma circulating tumor DNA (ctDNA) for predicting response to hyperthermic intraperitoneal chemotherapy (HIPEC), monitoring tumor burden, and predicting prognosis in patients with peritoneal carcinomatosis (PC).

**Experimental Design:**

In this observational study, 19 patients with PC were enrolled. Serial plasma ctDNA was analyzed using next-generation sequencing. The molecular tumor burden index (mTBI) was used to detect ctDNA, and concurrent changes in the dominant clone variant allele frequency (VAF) and common tumor markers were used as controls. The correlation between ascites and plasma ctDNA comutated genes was expressed by VAF. The overall response rate (complete response + partial response) after HIPEC was determined. Ascites progression-free survival (PFS) and overall survival (OS) were determined, and potential correlations between these outcomes and change in mTBI (△mTBI), change in sum-VAF (△sum-VAF), dominant close VAF, and tumor markers were assessed.

**Results:**

The overall response rate at 1 month after HIPEC was 100%. The △mTBI (r = 0.673; P = 0.023) and △sum-VAF (r = 0.945; P <0.001) were significantly positively correlated with ascites PFS; these correlations were stronger than those of the dominant clone VAF (r = 0.588; P = 0.057) and tumor markers in the same period (r =0.091; P = 0.790). Patients with a low baseline mTBI (<0.67) demonstrated significantly longer ascites PFS (P = 0.003; HR = 0.157; 95% CI: 0.046–0.540) and OS (P = 0.017; HR = 0.296; 95% CI: 0.109–0.804) than those with a high baseline mTBI (≥0.67). Consistent mutations were detected in plasma and ascites (r = 0.794; P = 0.001).

**Conclusion:**

A real-time serial plasma ctDNA assay accurately reflected tumor burden. The △mTBI and △sum-VAF can be used as predictors of HIPEC efficacy in patients with PC. A high baseline mTBI may be a negative risk factor for prognosis.

## Introduction

Gastric cancer, colorectal cancer, ovarian cancer, peritoneal false myxoma, peritoneal malignant mesothelioma, and the local progression of primary peritoneal carcinoma can lead to the development of peritoneal surface tumors, a condition usually referred to as peritoneal carcinomatosis (PC). The prognosis for affected patients is poor, with a median survival of approximately 6 months (1–3). Patients with this condition often receive palliative treatment.

As research regarding the biological behavior of tumors has expanded and improvements have been made in treatment technologies, the understanding of PC has also changed. Historically, PC has been considered widespread metastasis, but the condition is now considered a local disease and is treated accordingly. Since hyperthermic intraperitoneal chemotherapy (HIPEC) was first used to treat peritoneal pseudomyxoma in 1980 (4), this treatment has been gradually popularized for the treatment of patients with multiple malignant tumors with peritoneal metastasis such as PC.

Currently, the extent of PC involvement is usually quantified using the PC index (PCI) partition counting method (5). Studies have shown that the PCI is closely related not only to the long-term survival rate but also to the efficacy of HIPEC in patients with PC (6). However, to calculate the PCI, the size of tumor nodules in each region must be determined by completely exposing the abdominal viscera and parietal peritoneal surface, making this a complex and traumatic procedure. A less invasive technique for determining the extent of disease is needed.

Circulating tumor DNA (ctDNA) is released from tumor cells into the blood, carrying the mutations found in the original tumor (7). In recent years, ctDNA has been evaluated as a novel biomarker for liquid biopsy for both diagnosis and prognostic evaluation in patients with cancer (8, 9), offering a new technique for molecular diagnosis and disease monitoring (10). Compared with tissue biopsy, ctDNA liquid biopsy is a less invasive method for analyzing the entire cancer genome mutation spectrum (11, 12). In addition to blood, ctDNA can be detected in a variety of body fluids, including urine, cerebrospinal fluid, pleural fluid, saliva, and ascites. Among these materials, ctDNA in ascites may provide additional information that has not been detected in plasma ctDNA in various tumors (13–15). However, the role of ctDNA in plasma and ascites from patients with PC has not been fully elucidated.

In this study, we performed next-generation sequencing (NGS) of ctDNA from the plasma and ascites of 19 patients with PC both before and after they underwent HIPEC treatment. The primary objective of this study was to determine the accuracy of plasma ctDNA as a predictor of the efficacy of HIPEC and to compare its predictive accuracy with that of various tumor markers. The secondary objective was to elucidate the consistency of mutations between the two samples (plasma and ascites ctDNA).

## Materials and Methods

### Patient Population and Treatment

Patients with PC treated with HIPEC at Changzhou Second People’s Hospital affiliated with Nanjing Medical University in Jiangsu Province, China, between November 2018 and January 2020 were eligible for study participation. The inclusion criteria were as follows: PC confirmed by cytology and pathology; Eastern Cooperative Oncology Group (ECOG) score 0-2; no treatment with systemic chemotherapy or local radiotherapy in the previous month; and no history of HIPEC treatment. The main exclusion criteria were as follows: presence of any serious cardiopulmonary disease; presence of significant liver and kidney insufficiency; presence of acute intestinal obstruction; and presence of other contraindications for HIPEC. Based on these criteria, 19 patients were included in the final study population. All patients signed a written consent form before the study began. The research protocol was approved by the institutional Ethics Committee of Changzhou Second People’s Hospital.

Each of the study patients was treated with HIPEC 4 times within a 2-week time period, with each treatment session lasting 90 minutes. The perfusion drugs used were raltitrexed (2.5 mg/m^2^; 1 treatment session), docetaxel (25 mg/m^2^; 1 treatment session), and oxaliplatin (40 mg/m^2^; 2 treatment sessions). The agents were infused at an intraperitoneal temperature of 43°C.

### Sample Collection and Processing

Peripheral blood and ascites samples were collected from each patient within 1 week before HIPEC, and peripheral blood was collected within 1 week after HIPEC (all patients’ ascites resolved after HIPEC). Ascitic fluid (≥10 mL) was centrifuged at 600 g for 10 min to separate the supernatant and the precipitate. Peripheral blood (>10 mL) was collected in cell-free DNA (cfDNA) blood collection tubes (Streck, Omaha, NE, USA) at room temperature. Within 4 hours, plasma was separated from the blood samples by centrifugation at 1,600 × g at 4°C for 10 min; the supernatants were then centrifuged at 16,000 × g at 4°C for 10 min. Plasma, peripheral blood lymphocytes, ascites supernatant, and ascites precipitate were retained and stored at –80°C before extraction of cfDNA and genomic DNA (gDNA).

### DNA Extraction, Library Construction, and NGS

cfDNA from plasma and ascites supernatants was purified using the Circulating Nucleic Acid Kit (Qiagen, Hilden, Germany) following the manufacturer’s protocol. The DNA concentration was measured using the Qubit dsDNA HS (High Sensitivity) assay kit in the Qubit fluorometer (Invitrogen; Thermo Fisher Scientific, Inc., Waltham, MA, USA). To test the DNA integrity, 200 ng of extracted DNA was loaded onto the 1% agarose gel with λ-Hind III digest DNA marker (Takara Biotechnology Co., Ltd., Dalian, China). The DNA samples that were longer than the second largest bonds (9,416 bp) of λ-Hind III digest DNA marker were considered integrated samples and used for subsequent analysis.

Tumor DNA indexed NGS libraries were prepared using the DNA Library Preparation Kit for MGISeq-2000 (BGI, Shenzhen, China). All libraries were hybridized to custom-designed biotinylated oligonucleotide probes (IDT, Coralville, IA, USA) covering 382 genes. DNA sequencing was performed using the MGISeq-2000 Sequencing System (BGI, Shenzhen, China) per the manufacturer’s guideline; this generated 3 GB of data from tumor DNA.

### PyClone and mTBI Analysis

PyClone was used to analyze the population structure of ctDNA clones collected longitudinally from each patient. Information about single nucleotide variants (SNV) and copy number variations (CNVs) was used as input for PyClone analysis (16, 17), which was performed using 20,000 iterations and default parameters. The cluster with the greatest mean variant allele frequency (VAF) value was identified as the dominant clonal cluster, and the mutations in this cluster were considered clonal mutations. Other clusters and mutations were considered subclones. The sum-VAF was calculated as the total of VAF in each patient. Change in the VAF of the dominant clone mutation before and after HIPEC was calculated as follows: △VAF = baseline VAF – post-HIPEC VAF.

The molecular tumor burden index (mTBI) was determined using the mean VAF of clonal mutations. The change in mTBI was calculated as follows: △mTBI = baseline mTBI – post-HIPEC mTBI. This value was used to describe the degree to which the mutations of a patient were cleared after treatment.

### HIPEC Efficacy Evaluation

The curative efficacy of HIPEC was evaluated according to the 1981 WHO evaluation standard of curative effect, with brightness mode ultrasound (B-US) or computed tomography (CT) performed every 6 to 8 weeks during follow-up to detect the amount of ascites. Complete response (CR) was defined as the complete resolution of ascites lasting for >4 weeks; partial response (PR), a decrease of >50% in ascites lasting for >4 weeks; stable disease (SD), a decrease of <50% in ascites or no change in ascites; and progressive disease (PD), an increase in ascites. The objective response rate was defined as complete response + partial response.

### Statistical Analysis

Ascites progression-free survival (ascites PFS) and overall survival (OS) were the clinical endpoints in this study. Ascites PFS and OS were defined as the time from the start of treatment to ascites progression or death, respectively, or the time to last follow-up. The cutoff date for the analysis was December 31, 2020; all patients who had no progression in ascites or were alive as of then were censored on that date unless their date of last follow-up was earlier, in which case that date was used for censoring.

Data were reported as median and 95% confidence intervals (95% CIs) or ranges. The Wilcoxon rank-sum test was used to detect differences in mTBI changes before and after HIPEC treatment. Spearman correlation analysis was used to test the correlation between two variables (eg, △mTBI and ascites PFS). Ascites PFS and OS were analyzed using the Kaplan–Meier method. IBM SPSS software (V23.0) and GraphPad Prism (V8.2) were used for analysis. P < 0.05 was defined as statistically significant.

## Results

### Patient Characteristics, Patient Outcomes, and Mutation Detection

The clinical characteristics of the 19 study patients are shown in **Table 1**. The mean age was 57 years, and 73.68% of the patients were female. The following primary tumors were observed: ovarian cancer, 8 (42.11%); gastric cancer, 5 (26.31%); appendix cancer, 2 (10.53%); rectal cancer, 1 (5.26%); endometrial cancer, 2 (10.53%); and pancreatic cancer, 1 (5.26%) (**Table 2**). Four patients (21.06%) had only peritoneal metastasis; the remaining 15 patients (78.94%) had metastases in other organs, as well.

**Table 1:** Clinical characteristics of 19 patients with peritoneal carcinomatosis.

Characteristic	Value
Age (y)	
Mean ± SD	57.63 ± 9.67
Sex, no. (%)	
Male	5 (26.32)
Female	14 (73.68)
ECOG performance status, no. (%)	
0-1	14 (73.68)
2	5 (26.32)
Tumor differentiation, no. (%)	
Well/Moderate	10 (52.63)
Poor	9 (47.37)
Stage, no. (%)	
IIIB	1 (5.26)
IIIC	4 (21.06)
IV	14 (73.68)
Metastasis, no. (%)	
Peritoneum only	4 (21.06)
Peritoneum and other organs	15 (78.94)

ECOG, Eastern Cooperative Oncology Group; HIPEC, hyperthermic intraperitoneal chemotherapy.

**Table 2:** Primary tumor types and sample collection for 19 patients with peritoneal carcinomatosis.

Patient ID	Age (y)	Sex	Primary tumor	Baseline plasma sample	Post-HIPEC plasma sample	Baseline ascites sample
P01	75	Male	Gastric cancer	(+)	(+)	(+)
P02	45	Female	Appendix cancer	(+)	(+)	(+)
P03	50	Female	Ovarian cancer	(+)	(−)	(+)
P04	48	Female	Ovarian cancer	(+)	(+)	(+)
P05	70	Male	Gastric cancer	(+)	(+)	(+)
P06	67	Female	Gastric cancer	(+)	(+)	(+)
P07	61	Female	Ovarian cancer	(+)	(+)	(+)
P08	69	Female	Endometrial cancer	(+)	(−)	(+)
P09	49	Female	Ovarian cancer	(+)	(+)	(+)
P10	53	Female	Gastric cancer	(+)	(+)	(+)
P11	53	Female	Rectal cancer	(+)	(+)	(+)
P12	64	Male	Pancreatic cancer	(+)	(−)	(+)
P13	51	Female	Ovarian cancer	(+)	(+)	(−)
P14	69	Female	Ovarian cancer	(+)	(+)	(−)
P15	57	Male	Gastric cancer	(+)	(+)	(−)
P16	47	Male	Appendix cancer	(+)	(+)	(−)
P17	68	Female	Endometrial cancer	(+)	(−)	(−)
P18	48	Female	Ovarian cancer	(+)	(+)	(−)
P19	51	Female	Ovarian cancer	(+)	(−)	(−)

HIPEC, hyperthermic intraperitoneal chemotherapy.

The median follow-up among study patients was 9 months (range, 6–14 mo). At 1 month after HIPEC, the ORR was 100%. The median ascites PFS was 5.13 months (95% CI: 3.75–6.15 mo), and the median OS was 9.23 months (95% CI: 7.22–10.59 mo).

Information about the samples collected is shown in **Table 2**. Among the 19 patients, ascites was collected from 12 patients at baseline (samples were not collected from 5 patients, and 2 patients had no ascites at baseline). After HIPEC, ascites were resolved in all patients, so samples could not be collected. Baseline plasma samples were collected from all 19 patients, and post-HIPEC serial plasma samples were collected from 14 patients. A total of 37 functional mutations were detected in 7 of the 12 ascites samples collected at baseline (detection rate, 58.33%). A total of 51 functional mutations were detected in 10 of the 14 plasma samples collected at baseline (detection rate, 71.43%). A total of 38 functional mutations were detected in 8 of the 14 plasma samples collected after HIPEC (detection rate, 57.14%). In addition, 27 CNVs were detected in ascites samples and 4 CNVs were detected in baseline plasma samples. The most common CNVs were in *MYC*, *RECQL4*, *KRAS*, and *EGFR*.

### △mTBI as a Predictor of HIPEC Efficacy

**Figure 1:**
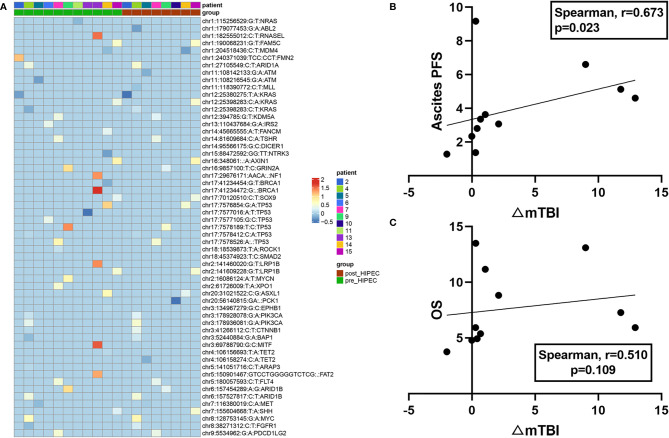
Plasma mutation landscapes and associations with prognosis. **(A)** Mutational landscape of 11 patients with PC. Green represents the patient’s gene mutation at baseline, and red represents the patient’s gene mutation after HIPEC (top). The gene symbol and VAF of each gene are shown (right). **(B)** Correlation between mTBI changes (△mTBI) and ascites PFS. **(C)** Correlation between mTBI changes (△mTBI) and OS.

Among the 14 patients with serial plasma samples, no gene mutations were detected in the plasma samples obtained from 3 patients (P1, P16, and P18) at baseline and after HIPEC, so the mTBI of the two nodes was 0 for these patients. Thus, a gene mutation heatmap based on serial plasma samples from the remaining 11 patients is presented (**Figure 1A**). For these 11 patients, the change in mTBI from baseline to after HIPEC (△mTBI) was significant (Wilcoxon, P = 0.026). In these patients, the median ascites PFS was 3.35 months (95% CI: 2.34–5.13 mo) and the median OS was 5.93 months (95% CI: 4.93–11.17 mo). There was a significant positive correlation between △mTBI and ascites PFS (Spearman r = 0.673; P = 0.023) (**Figure 1B**). The △mTBI was moderately positively correlated with OS (Spearman r = 0.510; P = 0.109) (**Figure 1C**), but the correlation was not significant.

### Baseline mTBI as a Prognostic Factor

**Figure 2:**
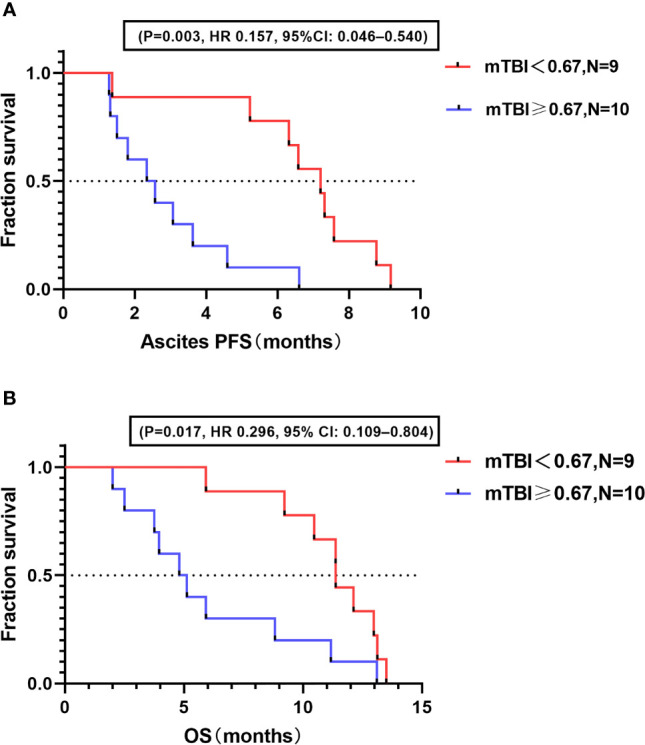
Correlation between baseline mTBI and prognosis. **(A)** Kaplan–Meier analysis for ascites PFS in high and low mTBI groups. **(B)** Kaplan–Meier analysis for OS in high and low mTBI groups.

The baseline median mTBI for the 19 study patients was 0.67. Based on this value, the patients were divided into a high mTBI group (≥0.67; n = 10) and a low mTBI group (<0.67; n = 9). Survival analysis demonstrated that ascites PFS was longer in the low mTBI group (median ascites PFS, 7.20 mo; 95% CI: 5.23–8.77 mo) than in the high mTBI group (median ascites PFS, 2.46 mo; 95% CI: 1.50-4.12 mo; HR = 0.157; 95% CI: 0.046–0.540; P = 0.003) (**Figure 2A**). Similar results were observed for OS, with patients in the low mTBI group having a longer OS (median OS, 11.38 mo; 95% CI: 9.23–13.13 mo) than those in the high mTBI group (median OS, 4.97 mo; 95% CI: 3.24-10.00 mo; HR = 0.296; 95% CI: 0.109–0.804; P = 0.017) (**Figure 2B**).

### △VAF and △ Tumor Markers as Predictors of HIPEC Efficacy

**Figure 3:**
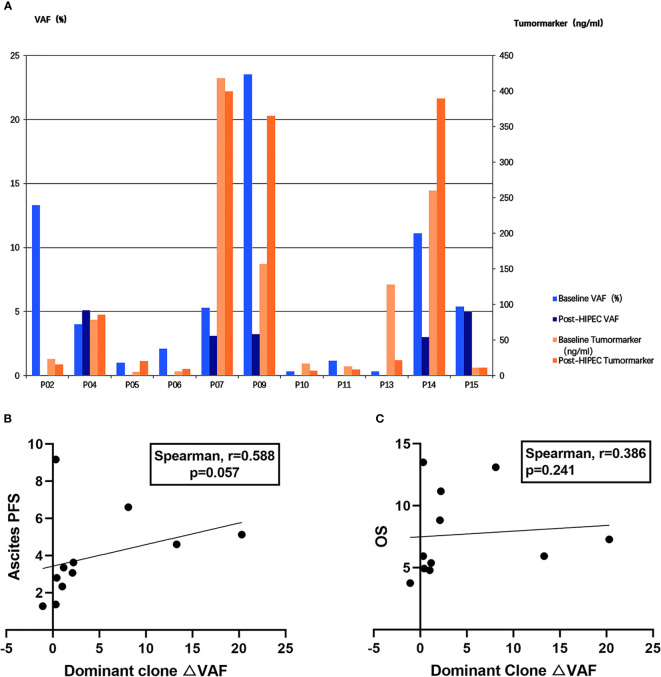
Comparison of correlation intensity between △mTBI, △VAF, and △ tumor markers and prognosis. **(A)** △VAF and △ tumor markers in 11 patients before and after HIPEC. VAF is displayed on the left y-axis. Tumor marker is displayed on the right y-axis. Patient IDs are displayed on the x-axis. **(B)** Correlation between dominant clone changes (△VAF) and ascites PFS. **(C)** Correlation between dominant clone changes (△VAF) and OS.

To assess the correlation between efficacy and changes in VAF and tumor markers, we examined changes in the primary clonal VAF and the most significant tumor markers (eg, CEA, CA125) in 11 patients (**Figure 3A**). Four patients (P5, P6, P9, and P14) were found to have inconsistent trends in VAF and tumor markers during the same period. The △VAF was moderately positively correlated with ascites PFS (Spearman r = 0.588; P = 0.057) (**Figure 3B**), and slightly positively correlated with OS (Spearman r = 0.386; P = 0.241) (**Figure 3C**), but these results were not significant. There was no correlation between the △ tumor markers and ascites PFS (Spearman r = 0.091; P = 0.790) or OS (Spearman r = 0.287; P = 0.396). Both of these factors were less correlated with efficacy than mTBI.

### △sum-VAF as a Prognostic Factor

**Figure 4:**
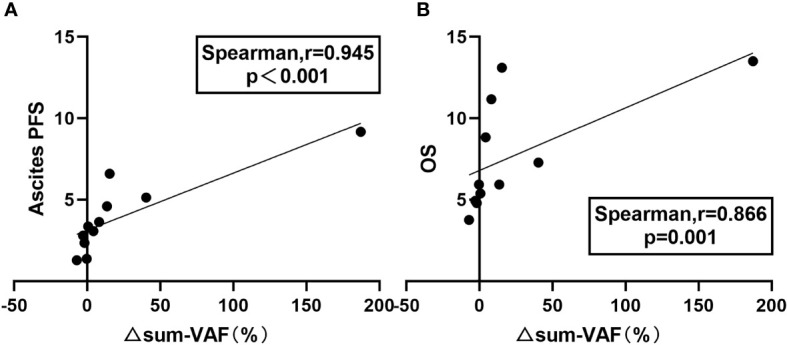
Relationship between △sum-VAF and prognosis. **(A)** Correlation between △sum-VAF and ascites PFS. **(B)** Correlation between △sum-VAF and OS.

Because the highest VAF does not accurately represent the overall tumor burden, we also analyzed the correlation between the change in sum of VAF (△sum-VAF = baseline sum-VAF – post-HIPEC sum-VAF) and ascites PFS and OS. There was a significant positive correlation between △sum-VAF and ascites PFS (Spearman r = 0.945; P < 0.001) (**Figure 4A**) and between △sum-VAF and OS (Spearman r = 0.866; P = 0.001) (**Figure 4B**).

### Consistency Analysis for Ascites and Plasma ctDNA

**Figure 5:**
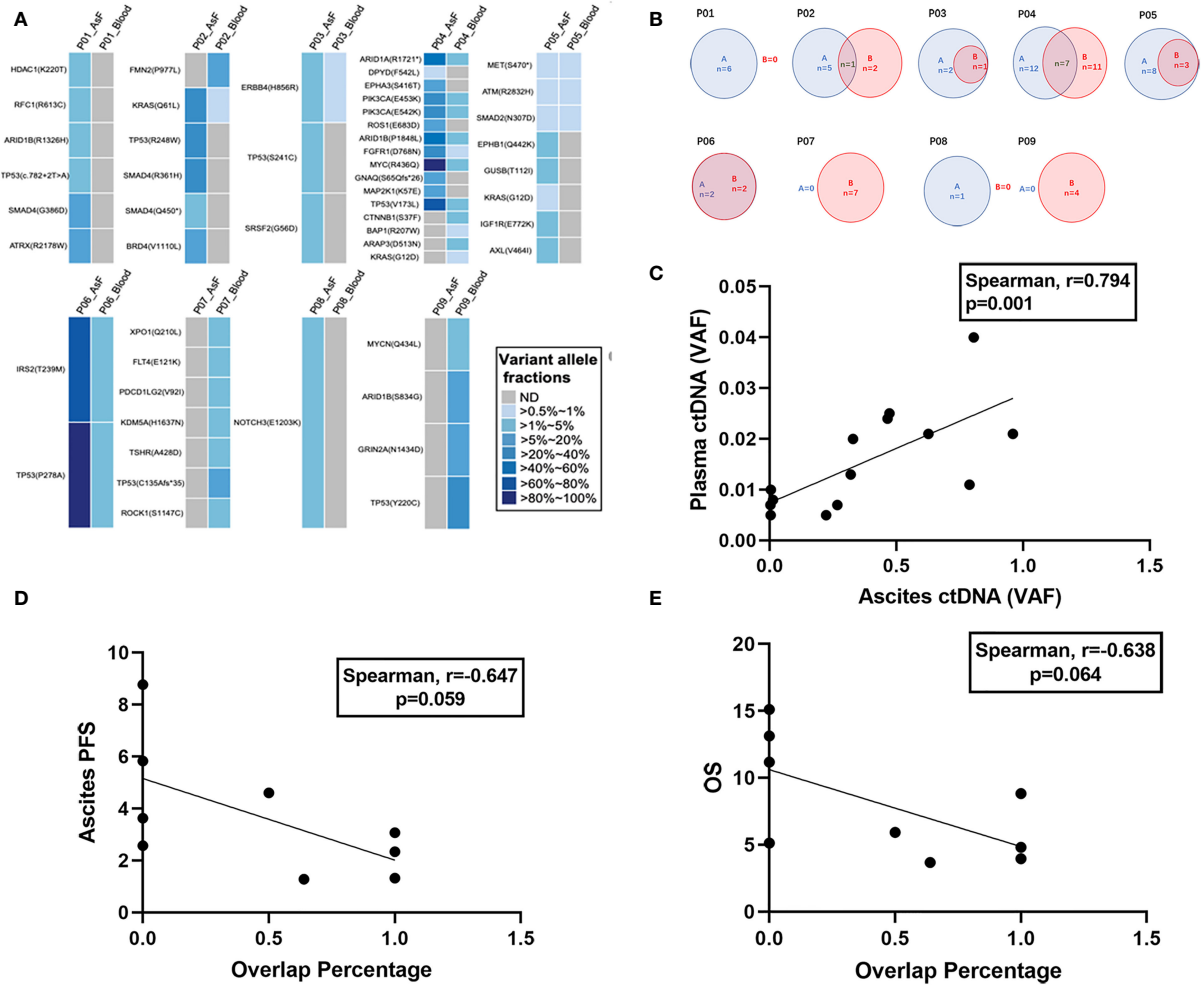
Mutational concordance between baseline ascites ctDNA and plasma ctDNA. Relationship between ascites entry rate and prognosis. **(A)** Comparison of VAF in plasma and ascites at baseline. **(B)** Numbers of somatic mutations codetected in ascites and plasma ctDNA are shown in blue and red, respectively. **(C)** Comparison of VAFs of the shared mutations between ascites and plasma ctDNA. The VAFs are shown on the x-axis and y-axis. **(D)** Correlation between ascites entry rate and ascites PFS. **(E)** Correlation between ascites entry rate and OS.

Among the 12 patients with baseline ascites samples and both baseline and post-HIPEC plasma samples, 3 patients (P10, P11, and P12) had negative test results (no mutation was detected) for both samples. For the remaining 9 patients, VAF values for the ascites and plasma samples were compared (**Figure 5A**). No mutation was detected in the plasma samples of 2 patients (P1 and P8), and no mutation was detected in the ascites samples of 2 patients (P7 and P9). A common mutated gene (**Figure 5B**) was observed in both samples from 5 of the 9 patients. In addition, there was a significant positive correlation between the ascites and plasma comutation gene VAF (Spearman r = 0.794; P = 0.001) (**Figure 5C**).

We calculated the number of comutations/the total number of mutations in plasma samples as the rate of ascites entry and assessed whether there was a correlation with ascites PFS and OS. The ascites entry rate was negatively correlated with ascites PFS (Spearman r = –0.647; P = 0.059) (**Figure 5D**) and OS (Spearman r = –0.638; P = 0.064) (**Figure 5E**), but the correlation was not significant.

## Discussion

In this study, we sought to determine the clinical significance of ctDNA in patients with PC undergoing HIPEC. We found that dynamic ctDNA monitoring using a series of plasma samples can be helpful in predicting the efficacy of HIPEC, which is consistent with the results of previous studies (18–21). We also found that △mTBI and △sum-VAF were significantly more predictive of efficacy than changes in tumor markers and the dominant clone VAF. In addition, we demonstrated that the baseline mTBI may be an accurate predictor of prognosis in patients with PC. Finally, we found that the consistency between plasma and ascites ctDNA in patients with PC was high, further confirming the clinical value of ctDNA in ascites samples.

Previous studies have identified factors that may serve as predictors of HIPEC efficacy in patients with PC. For example, both Yarema et al. (22) and Manzanedo et al. (23) demonstrated that PCI was one such predictor. In a multivariate survival analysis of 90 patients with PC, Yonemura et al. (24) found that patients who had undergone complete cytoreductive surgery were the best candidates for HIPEC. Akiyama et al. (25) observed that HIPEC was more effective in patients with miliary PC than in those with nodular PC. Kiuchi et al. (26) found that the prognosis for patients with poorly differentiated adenocarcinoma or signet-ring cell carcinoma who were treated with HIPEC was better than that of patients who underwent surgery alone. Research has also shown that HIPEC performed after surgery is more effective than surgery plus systemic chemotherapy in patients with invasive and diffuse advanced gastric cancer (27). Many studies have demonstrated that HIPEC does not improve survival for PC patients with ascites, so ascites can be considered a negative prognostic factor for treatment with HIPEC (28–31). All of these predictors have disadvantages; for example, PCI can be calculated only *via* invasive examinations, and determinations of tumor pathological types lack timeliness and accuracy because of tumor heterogeneity.

In this study, we measured plasma ctDNA at baseline and after HIPEC and used mTBI to quantify the frequency of gene mutations. We found that a decrease in mTBI was significantly positively correlated with prognosis, and the change in mTBI value was more closely correlated with prognosis than that of the dominant clone VAF or changes in tumor markers. In addition, we found that the 3 patients with no mutations detected at baseline and after HIPEC had a good prognosis. Two patients had mTBI > 0 at baseline; in both patients, mTBI decreased to 0 after HIPEC, and 1 patient demonstrated the longest ascites PFS (9.17 mo) in the study. We also used the median baseline mTBI (0.67) to divide patients into low mTBI and high mTBI groups and found that patients in the low mTBI group had better ascites PFS and OS than those in the high mTBI group. A high baseline mTBI (≥0.67) may therefore be a negative prognostic factor for patients undergoing HIPEC. Assessment of the correlation between prognosis and △mTBI, △VAF, and △ tumor markers demonstrated that △mTBI was the most strongly correlated with prognosis. Finally, we analyzed △sum-VAF, which better represents overall tumor burden, and found that △sum-VAF is another significant predictor of prognosis. It is possible that combining this indicator with △mTBI may increase the overall prognostic accuracy for patients treated with HIPEC.

All of the enrolled patients were confirmed to have peritoneal metastasis, but 7 patients did not have baseline ascites samples, so we included only 12 patients in our baseline dual-sample (ascites + plasma) analysis. In this analysis, the detection rate of gene mutations in plasma samples at baseline (71.43%) was significantly higher than the rate in ascites samples (58.33%). Subsequently, we found that ascites samples were highly consistent with the plasma comutated gene VAF. These results support the clinical significance of ctDNA detection in the ascites of patients with PC and verify previous findings from Han et al. (15)

Our study had some limitations, including the small sample size. Although we chose the most appropriate statistical method for analysis, there may still have been statistical bias. We included 19 patients with PC who did not undergo primary tumor normalization. Different primary tumors have different treatment plans and survival periods; such heterogeneity is unavoidable. However, the most direct therapeutic effect of HIPEC is the removal of peritoneal lesions to control the occurrence and development of ascites. Therefore, the main focus of our study was whether ctDNA analysis could predict the control of ascites development with HIPEC. Secondly, we confirmed that the correlation between △mTBI and prognosis was stronger than that of changes in tumor markers and prognosis, but we did not test the multinode plasma ctDNA before disease progression as Han et al. (15) did, so we were not able to determine whether ctDNA could predict disease progression before images or tumor markers could. Finally, because this was a preliminary exploratory study, we analyzed only the correlation between ascites and comutated genes in plasma and found that ascites was highly correlated with the mutation frequency of comutated genes in plasma. Further research is needed regarding the correlation mechanism, including not only the correlation, but also any differences in gene mutations.

To date, only one clinical study regarding the ability of ctDNA to predict the efficacy of HIPEC has been published (32), and the mutation frequency of only a single site of the *KRAS* gene has been measured. The usefulness of NGS detection based on a single gene mutation site may be limited by patient-specific variations. However, in our study, a pan-oncogene panel was examined for both plasma and ascites samples, so more patient-specific somatic mutations or even new mutations could be captured, which may also have mitigated the effect of primary tumor heterogeneity (33). In addition, mTBI and sum-VAF were used to quantify the overall mutation frequency of ctDNA genes in plasma, further confirming the prognostic value of ctDNA detection in plasma and ascites samples.

## Conclusions

Despite the small number of patients enrolled in our study and the different types of primary tumors involved, our results clearly demonstrate that serial plasma ctDNA testing reflects the real-time tumor load in patients with PC. We found that △mTBI and △sum-VAF are strong predictors of HIPEC efficacy in patients with PC and are more strongly correlated with prognosis than △VAF and △ tumor markers. Our correlation analysis of the comutated gene VAF demonstrated a high consistency between plasma and ascites samples, suggesting that ctDNA detection using ascites samples from patients with PC may have a certain predictive value. Clinical studies with larger sample sizes and uniform primary tumor types are needed to confirm these results.

## Data Availability Statement

The datasets presented in this study can be found in online repositories. The names of the repository/repositories and accession number(s) can be found below: The Genome Sequence Archive under accession HRA001488 (https://bigd.big.ac.cn/gsa-human/browse/HRA001488).

## Ethics Statement

The studies involving human participants were reviewed and approved by the institutional ethics committee of Changzhou Second People’s Hospital. The patients/participants provided their written informed consent to participate in this study.

## Author Contributions

All authors listed have made a substantial, direct, and intellectual contribution to the work and approved it for publication.

## Conflict of Interest

The authors declare that the research was conducted in the absence of any commercial or financial relationships that could be construed as a potential conflict of interest.

## Publisher’s Note

All claims expressed in this article are solely those of the authors and do not necessarily represent those of their affiliated organizations, or those of the publisher, the editors and the reviewers. Any product that may be evaluated in this article, or claim that may be made by its manufacturer, is not guaranteed or endorsed by the publisher.
